# Cannabidiol induces antioxidant pathways in keratinocytes by targeting BACH1

**DOI:** 10.1016/j.redox.2019.101321

**Published:** 2019-09-05

**Authors:** Laura Casares, Víctor García, Martín Garrido-Rodríguez, Estrella Millán, Juan A. Collado, Adela García-Martín, Jon Peñarando, Marco A. Calzado, Laureano de la Vega, Eduardo Muñoz

**Affiliations:** a Jacqui Wood Cancer Centre, Division of Cellular Medicine, School of Medicine, University of Dundee, Dundee, Scotland, UK; b Innohealth Group, Madrid, Spain; c Instituto Maimónides de Investigación Biomédica de Córdoba (IMIBIC), Córdoba, Spain; d Emerald Health Biotechnology, Córdoba, Spain; e Departamento de Biología Celular, Fisiología e Inmunología, Universidad de Córdoba, Spain; f Hospital Universitario Reina Sofía, Córdoba, Spain

**Keywords:** Cannabidiol/ BACH1/ HMOX1/ NRF2/ Keratinocytes

## Abstract

Cannabidiol (CBD) is a major non-psychotropic phytocannabinoid that attracted a great attention for its therapeutic potential against different pathologies including skin diseases. However, although the efficacy in preclinical models and the clinical benefits of CBD in humans have been extensively demonstrated, the molecular mechanism(s) and targets responsible for these effects are as yet unknown. Herein we characterized at the molecular level the effects of CBD on primary human keratinocytes using a combination of RNA sequencing (RNA-Seq) and sequential window acquisition of all theoretical mass spectrometry (SWATH-MS). Functional analysis revealed that CBD regulated pathways involved in keratinocyte differentiation, skin development and epidermal cell differentiation among other processes. In addition, CBD induced the expression of several NRF2 target genes, with heme oxygenase 1 (HMOX1) being the gene and the protein most upregulated by CBD. CRISPR/Cas9-mediated genome editing, RNA interference and biochemical studies demonstrated that the induction of *HMOX1* mediated by CBD, involved nuclear export and proteasomal degradation of the transcriptional repressor BACH1. Notably, we showed that the effect of BACH1 on *HMOX1* expression in keratinocytes is independent of NRF2. *In vivo* studies showed that topical CBD increased the levels of HMOX1 and of the proliferation and wound-repair associated keratins 16 and 17 in the skin of mice. Altogether, our study identifies BACH1 as a molecular target for CBD in keratinocytes and sets the basis for the use of topical CBD for the treatment of different skin diseases including atopic dermatitis and keratin disorders.

## Highlights

• CBD upregulates *HMOX1* expression in keratinocytes *in vitro* and *in vivo.*
• CBD induces nuclear export and degradation of BACH1.
• CBD induces *HMOX1* expression in an NRF2-independent manner.
• CBD induces keratinocyte proliferation *in vivo.*

## 1 Introduction

The skin serves as a protective barrier against the environment and is constantly exposed to insults which can lead to the generation of reactive oxygen species (ROS). While low levels of ROS act as intracellular signalling messengers [1], high ROS levels lead to oxidative stress, which is deleterious, as it damages cellular macromolecules [2]. Oxidative stress-induced cell damage can lead to chronic inflammation and is involved in the pathogenesis of skin diseases, skin disorder and skin aging [3]. To counteract the harmful accumulation of ROS, healthy skin presents a battery of defence mechanisms including antioxidant and detoxification systems. Many of these systems are under the control of nuclear factor erythroid 2-like 2 (NRF2), the master regulator of the antioxidant responses. Under basal conditions, NRF2 is kept at low levels by the negative regulator KEAP1 [4,5], a substrate adaptor protein for the Cullin3-containing E3-ligase complex, that mediates NRF2 ubiquitination and subsequent degradation. Cell exposure to ROS or electrophiles impairs the NRF2-KEAP1 binding, leading to NRF2 stabilisation [6]. NRF2 then can translocate to the nucleus where it binds to antioxidant response elements (AREs) in the promoters of NRF2 target genes and activate their expression [7].

Among NRF2 target genes is the stress inducible enzyme HMOX1. This enzyme catalyses the rate-limiting reaction in heme catabolism and has important antioxidant and anti-inflammatory properties [8,9]. Due to these cytoprotective properties, *HMOX1* is highly induced by a variety of cellular stresses [10] (e.g. oxidative stress, UV irradiation, hydrogen peroxide, nitric oxide, heavy metals, phorbol esters, lipopolysaccharide and organic chemicals) and thus it is one of the more widely used markers for stress responses. Although *HMOX1* is positively regulated by NRF2, its expression is also negatively regulated by the transcription factor BTB And CNC Homology 1 (BACH1) [11,12]. Importantly, the primary event leading to *HMOX1* induction is the deactivation of BACH1 repression [13]. The current model postulates that BACH1 and NRF2 act together to control *HMOX1* expression with the negative effect of BACH1 being dominant over the positive effect of NRF2; thus, BACH1 must be displaced for NRF2 to access the *HMOX1* promoter and to induce its expression. Although *HMOX1* is the main and the best characterized BACH1 target gene, a subset of NRF2 target genes have also been suggested to be BACH1 target genes (i.e. *GCLC* and *SQSTM1*) [14,15]. In normal skin HMOX1 is expressed in the upper epidermis, with the most prominent HMOX1 expression in the granular layer [16]. Furthermore, HMOX1 expression is associated with keratinocytes differentiation, and it is expressed in differentiated keratinocytes [16].

Cannabidiol (CBD) is the best studied non-psychotropic phytocannabinoid and shows pleiotropic activities including antioxidant and anti-inflammatory effects [[17], [18], [19], [20]]. Based on these properties, CBD might have therapeutic utility in a number of conditions including skin disorders. Additionally, it has been suggested that cannabinoids can be an attractive therapeutic approach for the treatment of keratin disorder such as epidermolysis bullosa (EB), a rare genodermatoses caused by function-impairing mutations in keratins [21]. Interestingly a recent observational study using self-initiated topical CBD use in 3 patients with EB reported faster wound healing, less blistering, and amelioration of pain in all patients [22].

Previous reports have shown that in cell types other than skin cells, CBD induced the expression of *HMOX1* and other NRF2-dependent genes [23,24], however, the mechanism of action behind the effect of CBD on the NRF2 pathway is not known. In addition, to date no systematic analysis of the pathways regulated by CBD in keratinocytes has been performed. Herein we show for the first time that CBD is a BACH1 inhibitor and a weak NRF2 activator. Furthermore, we reveal that in keratinocytes, *HMOX1* expression is regulated by BACH1 in a NRF2-independent manner. Finally, we show that topical CBD application in mice increased the levels of HMOX1 in the epidermis (in agreement with our results in cells) and induced the expression of wound-repair and proliferation associated keratins 16 and 17.

## 2 Experimental procedures

### 2.1 Cell cultures

Normal human epidermal keratinocytes (NHEK) and Keratinocyte growth medium were purchased to Innoprot SL (Biscaia, Spain). The cells were cultured until confluence in 60 cm^2^ plates with medium change every 24–48 h. Then, the cells were cultured in fresh medium in the presence or the absence of CBD (10 μM) for 24 h. HaCaT cells used in the study have been validated by STR profiling and were routinely tested for mycoplasma. The generation of HaCaT-ARE-Luc cells has been described previously [25]. CRISPR-edited NRF2-KO HaCaT cells were produced by transfecting HaCaT cells with pLentiCRISPR-v2 (a gift from Dr Feng Zhang, Addgene plasmid #52961) containing a guide RNA directed against the exon 2 of the NFE2L2 locus (which encodes NRF2) (5′-TGGAGGCAAGATATAGATCT-3′). After 2 days of puromycin selection, cells were clonally selected by serial dilution, and positive clones were identified as previously described [26]. Control cells, referred as HaCaT wild type (HaCaT WT), are the pooled population of surviving cells transfected with an empty pLentiCRISPRv2 vector treated with puromycin. All cell lines were grown in RPMI containing 10% FBS at 37 °C and 5% CO_2_.

### 2.2 Antibodies and reagents

Antibodies recognizing BACH1 (F-9), anti-Lamin B2 (C-20), anti-Tubulin (TU-02) and anti-cytokeratin 16 (sc-53255) were obtained from Santa Cruz Biotechnology (Dallas, Texas, USA). anti-NRF2 (D1Z9C) was obtained from Cell Signaling Technology (Danvers, MA, USA), anti-HMOX1 (ab13243) and anti-cytokeratin 17 (ab109725) were obtained from Abcam (Cambridge, UK). HRP-conjugated secondary antibodies were obtained from Life Technologies (Carlsbad, California, USA). The siRNAs used as control or against BACH1, NRF2 and KEAP1 were the SMART pool: ON-Target Plus from Dharmacon (Lafayette, CO, USA). MG132 was obtained from Santa Cruz Biotechnology, and Leptomycin B was obtained from Cayman Chemicals (Ann Arbor, MI, USA). ^9^Δ-THC was purchase to Sigma Aldrich (San Louis, MI, USA) and other cannabinoids with a purity higher than 97% were obtained from Prof. Giovanni Appendino (University of Eastern Piedmont, Novara, Italy).

### 2.3 RNA-Seq

Total RNA was isolated from NHEK cells by Qiazol lysis reagent (Qiagen, Hilden, Germany) and purified with miRNeasy mini kit (Qiagen) following manufacturer's instructions. RNA was processed for high throughput sequencing using the Illumina TruSeq mRNA Sample Prep v2 kit (RS-122-2001). Transcriptome libraries were constructed by polyA purification. In brief, 1 μg of total RNA from each sample was used to construct a cDNA library, followed by sequencing on the Illumina HiSeq 2500 system with single end 50 bp reads and ~30 millions of reads per sample.

### 2.4 SWATH LC-MS/MS proteomics

Proteins were obtained by lysing NHEK cells in NP-40 buffer (50 mM Tris-HCl pH 7.5, 150 mM NaCl, 10% glycerol and 1% NP-40) supplemented with protease and phosphatase inhibitors and cleaned to remove contaminants by protein precipitation with TCA/acetone and solubilized in 50 μl of 0.2% RapiGest (Waters, Milford, MA, USA) in 50 mM ammonium bicarbonate. Protein extracts were subjected to trypsin digestion. RePLiCal iRT (PolyQuant GmbH, Bad Abbach, Germany) peptides were added to the peptide samples in order to calibrate retention times in the SWATH runs. Samples (1 μg) were analysed by LC-MS/MS using a data-independent SWATH acquisition using a Triple TOF 5600 + mass spectrometer (Sciex) coupled to a nLC system with a 2 h gradient (5%–30% ACN 0.1% formic acid, 300 nL/min, column: Thermo PepMap100 25 cm x 75 μm id). For building the spectral library, shotgun data-dependent acquisition runs (top 65 method) were performed on the same LC-MS equipment and gradient.

### 2.5 Transcriptomic and proteomic data analysis

RNA-Seq reads were pre-processed with Trimmomatic (v0.36) [27] and aligned to human genome assembly hg38 using HISAT2 (v2.1.0) [28]. Then, counts per gene were obtained with featureCounts (v1.6.1) [29] and the differential expression analysis was carried out using DESeq2 (v1.20.0) [30] excluding those genes with less than 15 counts across all samples. For proteomics, proteins and peptides were identified from the DDA shotgun runs using ProteinPilot (v5.0) and a concatenated target-reverse decoy SwissProt human protein database. A spectral library was built using the MS/MS ALL with SWATH Acquisition MicroApp (v2.0) using the peptides that showed up in the database search with a confidence score above 99%. The library-assisted targeted data extraction of the fragment ion chromatogram traces from the SWATH runs, and the retention time calibration, was performed by PeakView (v2.1) using the MS/MSALL with SWATH Acquisition MicroApp (v2.0), using a 1% FDR threshold and 50 ppm of tolerance. MarkerView (v1.2.1, Sciex) was used for signal normalization. Differential abundance analysis was performed by applying a Welch Two Sample T-Test to compare normalized SWATH areas between groups. For the functional analysis, genes with an adjusted P < 0.05 and an absolute fold change >2 and proteins with an adjusted P < 0.05 and an absolute fold change >1.5 were selected to perform an over-representation analysis. For this, the EnrichR tool was employed using Gene Ontology (Biological Process) terms, Wikipathways and transcription factors from JASPAR [31]. Finally, a gene set enrichment analysis was carried out for transcriptomic data with the fgsea (v1.9.7) package, using the same gene sets and pre-ranking the whole gene list by the log2 transformed fold change. All the P values from the different analyses were adjusted to control the false discovery rate (FDR) using the Benjamini and Hochberg approach [32]. RNA-seq data have been deposited in the Gene Expression Omnibus (GEO) databank with the dataset identifier GSE131565. The mass spectrometry proteomics data have been deposited to the ProteomeXchange Consortium via the PRIDE [33] partner repository with the dataset identifier PXD013956.

### 2.6 Quantitative real time PCR (rt-qPCR)

RNA was extracted using RNeasy kit (Qiagen). 500 ng of RNA per sample was reverse-transcribed to cDNA using Omniscript RT kit (Qiagen) supplemented with RNase inhibitor according to the manufacturer's instructions. Resulting cDNA was analysed using TaqMan Universal Master Mix II (Life Technologies, Carlsbad, CA, USA). Gene expression was determined using an Applied Biosystems 7300 Real-Time PCR system by the comparative ΔΔCT method. All experiments were performed at least in triplicates and data were normalized to the housekeeping gene HPRT1. The primers used are listed in Supplementary Table S1.

### 2.7 siRNA cell transfections

On the day prior to transfection, cells were plated to the required cell density (70–90% confluency). The siRNA and Lipofectamine RNAiMAX (Invitrogen, Carlsbad, CA, USA) were individually diluted in Optimem (Life Technologies) and incubated for 10 min at room temperature. Diluted siRNA was added to the diluted Lipofectamine solution (1:1 ratio) and further incubated for 15 min. The complex was added to the cells and incubated in a humidified incubator at 37 °C and 5% CO_2_ for 36 h prior treatment and lysis.

### 2.8 Cell lysis protocol and western blotting

Cells were washed and harvested in ice-cold phosphate-buffered saline (PBS) and lysed in either SDS buffer or RIPA buffer [50 mM Tris-HCl pH 7.5, 150 mM NaCl, 2 mM EDTA, 1% NP40, 0.5% sodium deoxycholate, 0.5 mM Na3VO4, 50 mM NaF, 2 μg/ml leupeptine, 2 μg/ml aprotinin, 0.05 mM pefabloc]. Cells directly lysed in SDS were boiled for 2 min, sonicated and boiled again for another 5 min. Cells lysed in RIPA buffer were sonicated and lysates were cleared by centrifugation for 15 min at 4 °C. Protein concentration was established using the BCA assay (Thermo Fisher Scientific, Waltham, MA, USA). Supernatant was mixed with SDS sample buffer and boiled for 5 min. Equal amounts of protein were separated by SDS-PAGE, followed by semidry blotting to a polyvinylidene difluoride membrane (Thermo Fisher Scientific). After blocking of the membrane with 5% (w/v) TBST non-fat dry milk, primary antibodies were added. Appropriate secondary antibodies coupled to horseradish peroxidase were detected by enhanced chemiluminescence using Clarity™ Western ECL Blotting Substrate (Bio-Rad, Hercules, CA, USA).

### 2.9 Subcellular fractionation (nuclear/cytoplasmic separation)

Cells were washed and harvested with ice-cold PBS. Pelleted cells were resuspended in 400 μl of low-salt buffer A (10 mM Hepes/KOH pH7.9, 10 mM KCL, 0.1 mM EDTA, 0.1 mM EGTA, 1 mM β-Mercaptoethanol). After incubation for 10 min on ice, 10 μl of 10% NP-40 was added and cells were lysed by gently vortexing. The homogenate was centrifuged for 10 s at 13,200 rpm in a microfuge. The supernatant representing the cytoplasmic fraction was collected and the pellet containing the cell nuclei was washed 4 additional times in buffer A, then resuspended in 100 μl high-salt buffer B (20 mM Hepes/KOH pH7.9, 400 mM NaCL, 1 mM EDTA, 1 mM EGTA, 1 mM β-mercaptoethanol). The lysates were sonicated and centrifuged at 4 °C for 15 min at 13,200 rpm. The supernatant representing the nuclear fraction was collected. Protease and phosphatase inhibitors were freshly added to both buffers.

### 2.10 Determination of heme

Heme levels were detected using the Hemin Assay Kit (MAK036, Sigma-Aldrich) according to manufacturer's instructions. Briefly, 2 × 10^6^ cells were homogenized in 4 vol of cold hemin assay buffer. Samples were centrifuged at 13000×*g* for 10 min at 4 °C to remove the cellular debris and diluted with hemin assay buffer. After adding the proper reaction mix and incubate for 10 min, absorbance was measure at 570 nm in a kinetic mode. Data were shown as amount of heme (fmole) using a hemin standard curve.

### 2.11 Luciferase assays

HaCaT-ARE-Luc cells were stimulated with either Sulforaphane (SFN) (5 μM) or with increasing concentrations of cannabinoids for 6 h. After the treatment the cells were washed twice in PBS and lysed in 25 mM Tris-phosphate pH 7.8, 8 mM MgCl_2_, 1 mM DTT, 1% Triton X-100, and 7% glycerol during 15 min at room temperature in a horizontal shaker. After centrifugation, luciferase activity in the supernatant was measured using a GloMax 96 microplate luminometer (Promega) following the instructions of the luciferase assay kit (Promega, Madison, WI, USA). Results are expressed in RLU over control untreated cells.

### 2.12 ROS determination

The intracellular accumulation of ROS was detected using 2',7-'dihydrofluorescein-diacetate (DCFH-DA). HaCaT cells (15 × 10^3^ cells/well) were cultured in a 96-well plate in DMEM supplemented with 10% FBS until cells reached 80% confluence. For induction of ROS the cells were treated with increasing concentrations CBD. For inhibition, the cells were pre-treated with CBD for 30 min and treated with 0,4 mM Tert-butyl-hydroperoxide (TBHP). Three hours later the cells were incubated with 10 μM DCFH-DA in the culture medium at 37°C for 30 min. Then, the cells were washed with PBS at 37°C and the production of intracellular ROS measured by DCF fluorescence was detected using the Incucyte FLR software, the data were analysed by the total green object integrated intensity (GCUxμm^2^xWell) of the imaging system IncuCyte HD (Sartorius, Göttingen, Germany). N-Acetyl cysteine (NAC) (15 mM) was used as a positive control that inhibited TPHP-induced ROS production.

### 2.13 Animal studies

Six-months old female BALB/cByJRj mice were obtained from Janvier Labs (Le Genest-Saint-Isle, France). Animals were housed in groups (control n = 3, CBD 0.1% n = 6, and CBD 1% n = 6) at 20–22 °C under constant conditions of light (14 h of light; lights on at 7:00 a.m.) and 40–50% relative humidity with free access to standard food and water. The back of the mice was shaved, and the animals were treated topically with vehicle (10% DMSO in propylene glycol), CBD 0.1%, 1% or 10% once a day for 5 days. At the end of the experiment, mice were euthanized, and mouse skin was collected, fixed in 4% paraformaldehyde and embedded in paraffin for subsequent histological analysis. All experiments were performed in accordance with European Union guideline and approved by the Animal Research Ethic Committee of The University of Córdoba (2014PI/025).

### 2.14 Histology and immunohistochemistry

Five-μm sections from mouse skin were deparaffinised with xylene and rehydrated with decreasing concentration of ethanol and water. Then, sections were stained with haematoxylin for 5 min, washed in running water and differentiated in acid ethanol for a few seconds. Once rinsed, sections were stained with eosin for 30 s and dehydrated with increasing concentration of ethanol. Finally, after a 2-min xylene bath, samples were mounted and analysed under the microscope. For IHC, antigen retrieval was performed by microwave heating for 5 min in citrate buffer (10 mM, Ph 6.0) for cytokeratins and HMOX1 antibodies. Neutralization of endogenous peroxidase was performed using EnVision FLEX-peroxidase blocking reagent (Agilent Dako, Glostrup, Denmark) for 10 min. Samples were blocked with 3% bovine serum albumin for 30 min, and the mouse-on-mouse staining protocol (Rodent Block M, RBM961L, Biocare Medical, Pacheco, CA, EEUU) was used for cytokeratin 16 mouse antibody. Then, tissue sections were incubated with primary antibodies overnight at 4 °C. Samples were incubated with EnVision FLEX + mouse/rabbit linker and EnVision FLEX/HRP (Agilent Dako), for 30 min at RT each. Finally, detection was performed using 3,3′-diaminobenzidine (DAB) chromogen and sections were counterstained with haematoxylin, dehydrated, mounted and analysed with a Leica DM2000 microscope. Pictures were taken with a Leica MC190 camera. The quantification of IHCs was performed measuring the stained area in the epidermis from more than 8 fields per condition with ImageJ software (Bethesda, MD, USA).

### 2.15 Statistical analyses

Most experiments were repeated 3–5 times with multiple technical replicates to be eligible for the indicated statistical analyses. Data were analysed using Graphpad Prism statistical package. All results are presented as mean ± SD unless otherwise mentioned. For animal studies, five-eight mice per group was the standard sample size as defined by statistical power analyses (80% power; p < 0.05) carried out using R packages. The investigators were not blinded to allocation during experiments and outcome assessment. When applicable, the differences between groups were determined by either one-way ANOVA or 2-way ANOVA. A P value of <0.05 was considered significant. *P ≤ 0.05, **P ≤ 0.01, ***P ≤ 0.001.

## 3 Results

### 3.1 Proteomics and transcriptomic analysis in primary keratinocytes treated with CBD

**Fig. 1:**
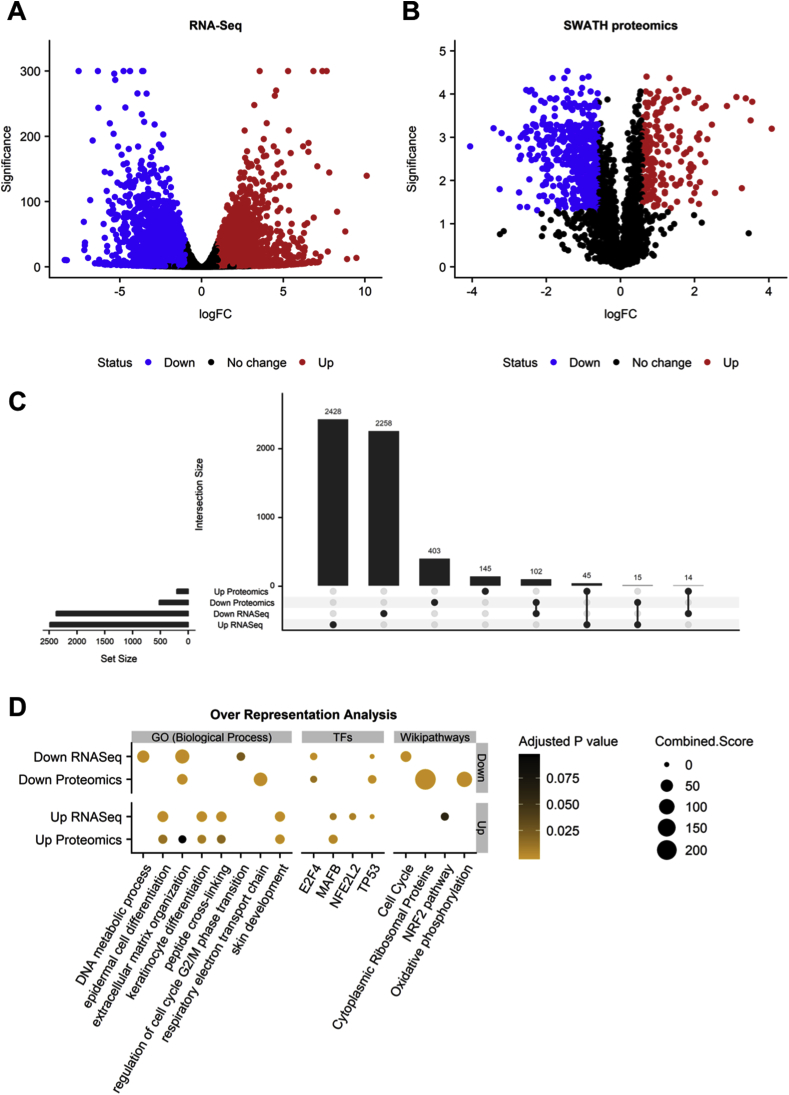
**Multi-omic analysis of the response of keratinocytes to CBD.** The transcriptomic and proteomic profiling of RNA and protein samples was carried out using RNA-Seq and LC-MS/MS, respectively. **(A, B)** Volcano plots showing the magnitude (log2 fold change) and significance (-log10 p value) of the changes in the transcriptomic and proteomic comparisons of CBD treated keratinocytes versus controls (n = 3 for RNA-Seq and n = 4 for proteomics). Every point represents a gene/protein and the colour indicates those surpassing the cut-off of an adjusted P value < 0.05 and an absolute fold change >2 (for genes) or > 1.5 (for proteins). For RNA-Seq, a small value (1e-300) was added to p values in order to avoid logarithms of zero at plotting. **(C)** Upset plot indicating the overlap between the sets of up or down regulated genes and proteins as a bar plot over a coincidence histogram. **(D)** Over-representation analysis results. The dot plot indicates with a point the significant over-representation of a given term, transcription factor or pathway in a group of up or down regulated genes/proteins (Fisher Exact Test adjusted P < 0.1). While the colour indicates the adjusted P value of the enrichment, the size of the point represents the enrichR combined score.

To obtain a first insight into the changes produced by CBD in epidermal cells, primary human keratinocytes were incubated with either solvent or CBD (10 μM) for 24 h and transcriptomic and proteomic changes were analysed through RNA-Seq and SWATH LC-MS/MS, respectively. From 17571 genes quantified the treatment with CBD altered the expression (adjusted P < 0.05 and absolute fold change > 2) of 4860 genes, of which 2374 were downregulated and 2486 upregulated (Fig. 1A). At the proteome level, 724 out of 2204 quantified proteins surpassed the cut-off (adjusted P < 0.05 and absolute fold change > 1.5). From them, 520 decreased and 204 increased their abundance (Fig. 1B). Additionally, 147 features were found to be significantly altered in the same direction at both levels when comparing the overlap between the mRNA and protein sets (Fig. 1C). Finally, to explore the functional impact of the observed changes, we performed an over-representation analysis of the different sets of up and down regulated genes and proteins using enrichR. We found several pathways enriched (adjusted P < 0.1) in the group of up-regulated features related with the skin biology as “epidermal cell differentiation”, “keratinocyte differentiation”, “skin development” and also transcription factors such as the NRF2 (“NFE2L2” and “NRF2 pathway”). On the other hand, we found terms and pathways that were enriched in the group of down-regulated genes and proteins as “extracellular matrix organization”, “DNA metabolic process” and “Cell Cycle” (Fig. 1D). This functional portrait of the transcriptomic and proteomic changes suggested that CBD may promote keratinocyte proliferation and differentiation, and upregulated genes and proteins implicated in skin development. However, the result that strongly caught our attention was the activation of the transcriptional activity of NRF2, given the importance of this antioxidant transcription factor in keratinocyte biology [34].

### 3.2 Validation of the NRF2 pathway as a target of CBD in keratinocytes

**Fig. 2:**
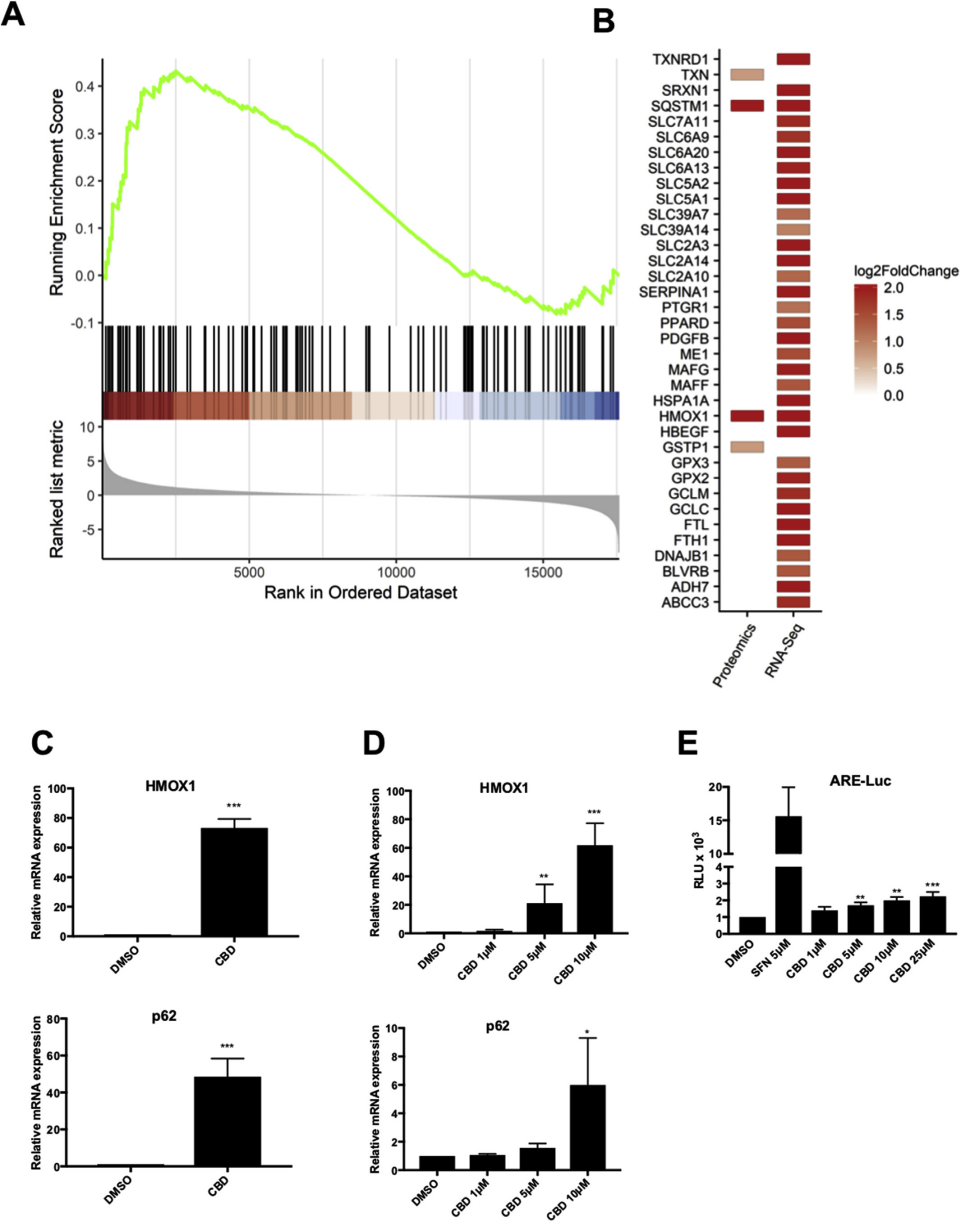
**Validation of the NRF2 pathway as a target of CBD in keratinocytes. (A)** Gene set enrichment analysis plot for the NRF2 signalling pathway transcriptomic changes. Black lines indicate the position of NRF2 genes in the pre-ranked gene list and the green line indicates the running enrichment score. **(B)** Magnitude of the changes for significantly up-regulated genes and proteins selected using the previously mentioned cut-offs in the CBD versus control comparison**. C)** Primary human keratinocytes were incubated with either DMSO or CBD (10 μM) for 24 h. The mRNA levels for *HMOX1* (*upper panel*) and *SQSTM1* (*p62)* (*lower panel*) were quantified using real-time PCR. The data were normalized using *HPRT1* as an internal control. Data represent means ± SD (n = 3) and are expressed relative to the DMSO sample. ***P ≤ 0.001**. D)** HaCaT cells were incubated with either DMSO or increasing concentration of CBD for 16 h. The mRNA levels for *HMOX1* (*upper panel*) and *SQSTM1* (*p62)* (*lower panel*) were quantified using real-time PCR as previously indicated (n = 3) *P ≤ 0.05, **P ≤ 0.01, ***P ≤ 0.001. **E)** HaCaT-ARE-Luc cells were treated with either SFN or CBD at the indicated concentrations for 6 h. Luciferase activity was measured in the cell lysates and expressed as RLU (x 10^4^). Data represent means ± SD (n = 4) and are expressed relative to untreated cells. **P ≤ 0.01, ***P ≤ 0.001.

In order to validate the data obtained from the RNA-Seq and mass spectrometry analysis, we selected candidates that were highly induced by CBD at both protein and mRNA levels (i.e SQSTM1, also known as p62, and HMOX1) (Fig. 2A–B), and we tested by qRT-PCR the effect of CBD on their expression in primary human keratinocytes (Fig. 2C) and in the immortalised human keratinocyte cell line HaCaT (Fig. 2D). Our results showed that in fact, CBD treatment induced the expression of the selected NRF2 target genes in both primary and immortalised human keratinocytes. Furthermore, using a HaCaT NRF2 reporter (ARE-luc) cell line we tested if CBD was also able to activate a synthetic reporter gene containing the ARE sequence from the NQO1 promoter fused to luciferase. Our results show that when compared with the potent NRF2 activator sulforaphane (SFN), CBD is a weak inducer of ARE-Luc (Fig. 2E).

### 3.3 CBD activates a subset of NRF2 target genes

**Fig. 3:**
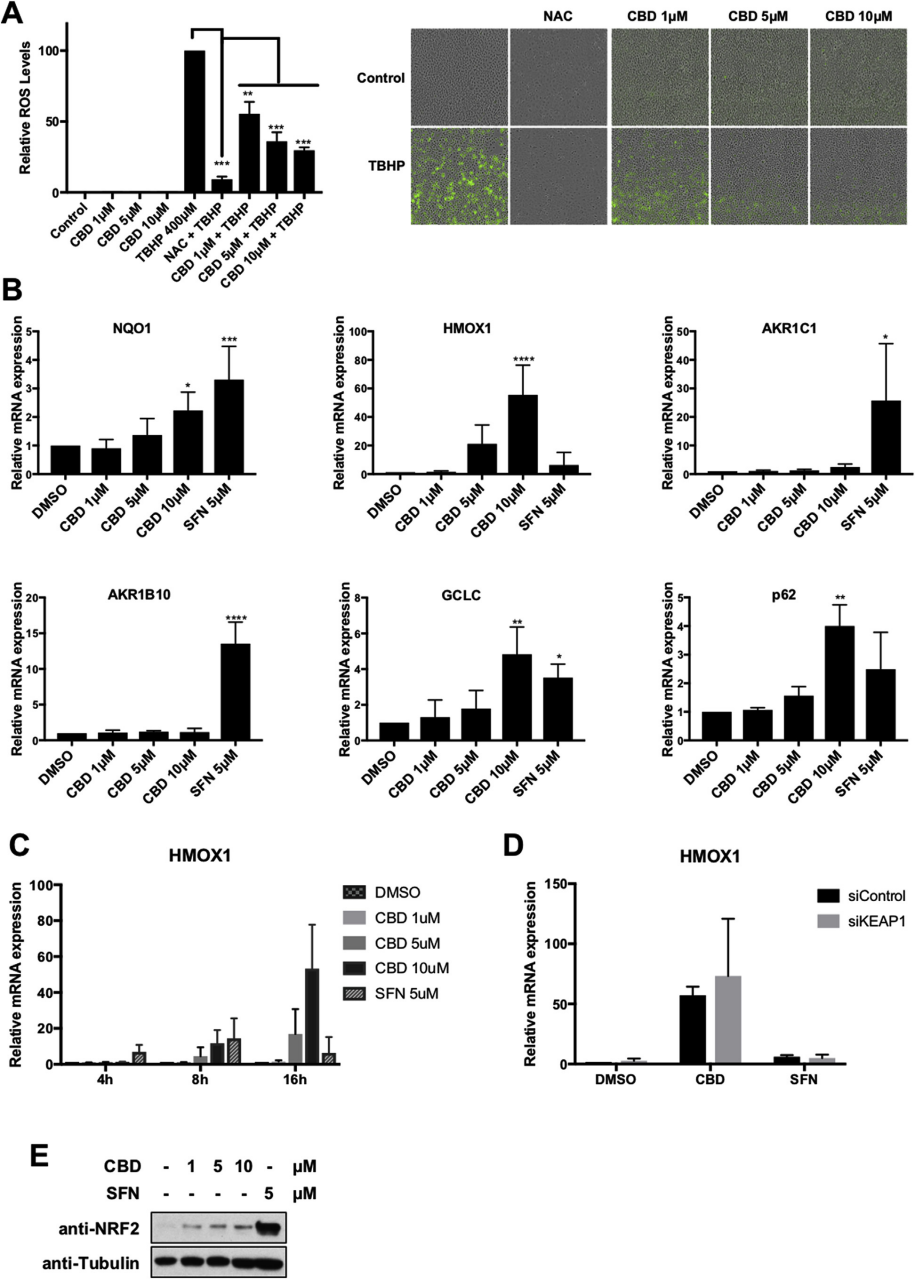
**CBD activates a subset of NRF2 target genes. A)** ROS detection in HaCaT labelled with DCFH-DA, HaCaT cells was treated as indicated and the detection and quantification of ROS (DCF fluorescence) measured by fluorescence microscopy (left panel). Data represent means ± SD (n = 5) and are expressed relative to control cells. **P ≤ 0.01, ***P ≤ 0.001. Representative pictures are shown in the right panel. **B)** HaCaT cells were incubated with either DMSO, SFN (5 μM), or increasing concentration of CBD for 16 h. The mRNA levels for the indicated genes were quantified using real-time PCR. The data were normalized using *HPRT1* as an internal control. Data represent means ± SD (n = 3) and are expressed relative to the DMSO sample. *P ≤ 0.05, **P ≤ 0.01, ***P ≤ 0.001, ****P ≤ 0.0001. **C)** HaCaT cells were incubated with either DMSO, SFN (5 μM), or increasing concentration of CBD for 4, 8 and 16 h. The mRNA levels for *HMOX1* were quantified using real-time PCR as previously indicated (n = 3). **D)** HaCaT cells were transfected with either siControl or siKEAP1. 36 h later cells were incubated with either DMSO, SFN (5 μM), or CBD (10 μM) for another 16 h. The mRNA levels for *HMOX1* were quantified as previously indicated (n = 3). **E)** HaCaT cells were incubated with either DMSO (−), CBD or SFN as indicated. Three hours later, cells we lysed, and levels of NRF2 and Tubulin were analysed by Western blot.

Compounds or drugs that activate the NRF2 pathway could act either directly by stabilising NRF2, or indirectly by inducing ROS which will consequently activate NRF2. Examples of the former are the natural compound sulforaphane [35] and the synthetic compound TBE31 [36]. As CBD, albeit weak, appeared to be an activator of NRF2, we wondered whether it was a direct activator, or if, as it has been suggested in the literature, CBD activates NRF2 indirectly by inducing ROS [37,38]. Our results showed that CBD, at the concentrations previously used, not only did not induce ROS, but it was able to reduce ROS levels induced by tBHP (tert-Butyl hydroperoxide) in a concentration-dependent manner (Fig. 3A). Secondly, as CBD was only weakly inducing the expression of an ARE-luc construct, we further tested whether CBD was a good NRF2 activator. To do so, we compared the effect of CBD and SFN on the expression of a panel of NRF2 target genes (Fig. 3B). Interestingly, CBD was equally or more potent than SFN at inducing the expression of a subset of NRF2 target genes (e.g. *HMOX1*, GCLC and *p62*), but dramatically less potent inducing the expression of other NRF2 target genes (i.e the aldo-ketoreductases AKR1B10 and AKR1C1). HMOX1 was the gene in which the effect of CBD was clearer and in which CBD was significantly more potent than SFN. To further characterise the differences between CBD and SFN we studied the kinetics of *HMOX1* induction in response to either CBD or SFN (Fig. 3C). Our data showed that in response to SFN, *HMOX1* induction peaked at 8 h after treatment (14-fold mean induction), returning to basal levels at 16 h. In contrast, CBD treatment maintained a high *HMOX1* expression at 16 h (53-fold mean induction) suggesting that CBD has a stronger or a more persistent effect on *HMOX1* than that of SFN. Notably, other cannabinoids such as ^9^Δ−THC, CBC or CBG were less potent in inducing *HMOX1*, and their acidic forms were completely inactive (Fig. S1A).

SFN, as most electrophilic compounds, acts via KEAP1, but as CBD is not electrophilic, we hypothesized that CBD might act in a KEAP1-independent manner. To test this hypothesis, we tested the effect of CBD on *HMOX1* expression in control cells or in cells were KEAP1 had been depleted. Our results showed that CBD was still able to induce the expression of *HMOX1* in KEAP1 knocked-down cells (Fig. 3D), demonstrating that the effect of CBD is independent of KEAP1. Interestingly, although KEAP1 knockdown greatly induced the expression of AKR1C1 (Fig. S1B), *HMOX1* basal levels were not significantly changed by KEAP1 knockdown, revealing that in this setting, activation of NRF2 by itself is not enough to induce *HMOX1* expression.

To further compare CBD and SFN, we directly assessed their effect on NRF2 protein levels. While SFN treatment strongly stabilised NRF2 protein levels, the effect of CBD was very mild in comparison (Fig. 3E). All together, these results suggest that a) the mechanism of action of both compounds is different; and b) the strong effect of CBD on *HMOX1* expression does not correlate with its weak effect on NRF2 stability, and therefore NRF2 activation is not likely the main mechanism responsible for CBD-mediated *HMOX1* activation.

### 3.4 CBD activates HMOX1 in a BACH1-dependent manner

**Fig. 4:**
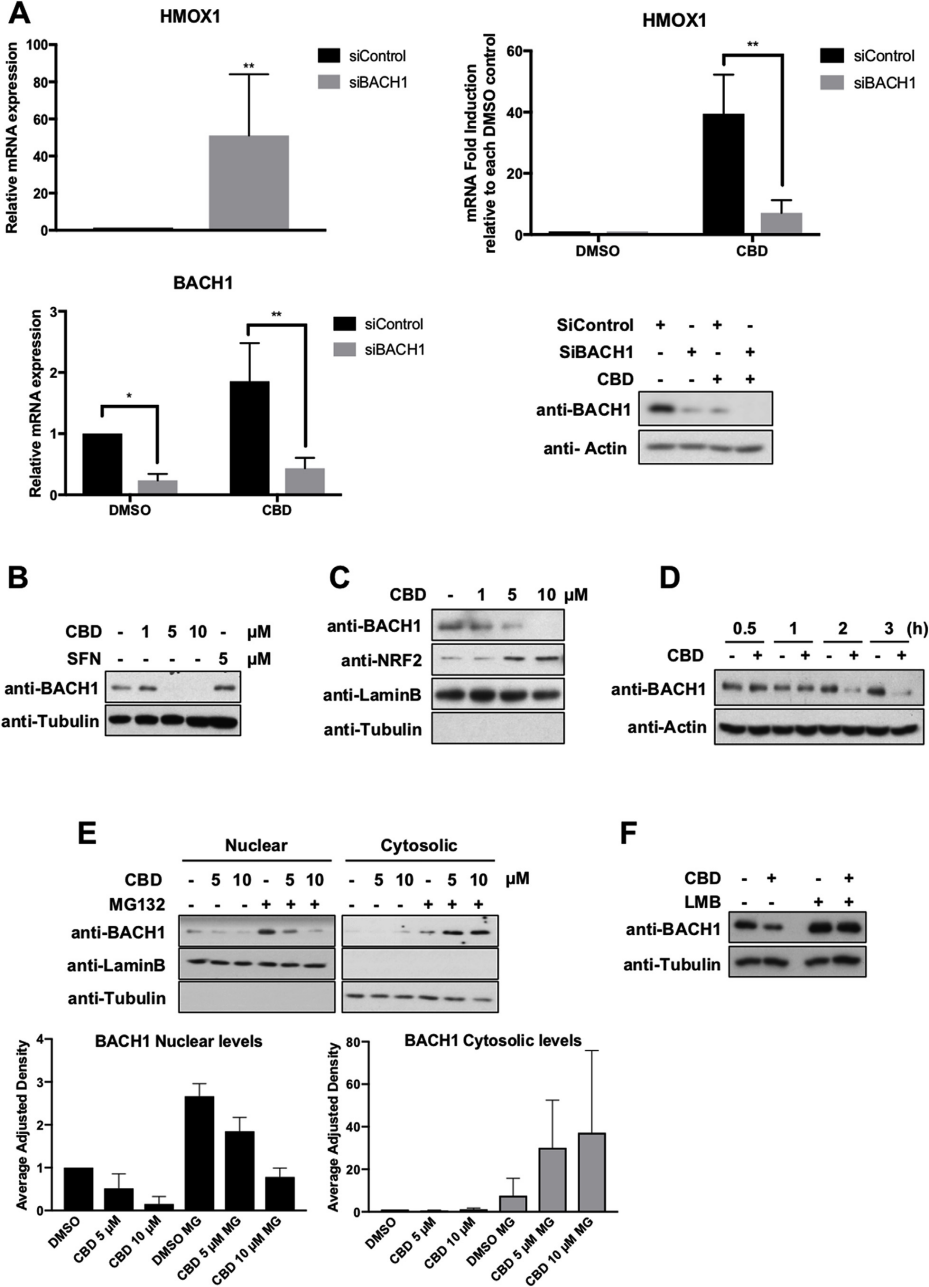
**CBD activates HMOX1 in a BACH1-dependent manner. A)** HaCaT cells were transfected with either siControl or siBACH1. 36 h later, cells were incubated with either DMSO or CBD (10 μM) for another 16 h. To measure the effect of BACH1 depletion on *HMOX1* expression, the mRNA levels for *HMOX1* in siControl and siBACH1 DMSO treated cells were quantified using real-time PCR as previously described *(upper left panel)*. To compare the *HMOX1* induction upon CBD treatment in each cell line, the levels of *HMOX1* in either siControl CBD or siBACH1 CBD samples were compared against the levels of *HMOX1* in siControl DMSO or siBACH1 DMSO samples respectively (*HMOX1* levels in DMSO samples were set in both cases as 1) *(upper right panel*). The CBD-mediated *HMOX1* induction in both cell lines was quantified by real-time PCR using *HPRT1* as an internal control. Data represent means ± SD (n = 3). To control for the efficiency of the knockdown, the mRNA levels *(lower left panel)* and protein levels *(lower right panel)* of BACH1 were analysed by real-time PCR and western blot respectively. *P ≤ 0.05, **P ≤ 0.01 . **B)** HaCaT cells were incubated with either DMSO (−), CBD or SFN as indicated. Three hours later, cells we lysed, and levels of BACH1 and Tubulin were analysed by Western blot. (These blots and the blots from Fig. 3E are from the same experiment, and thus share the same tubulin. For clarity's sake we decided to show them in two different subfigures). **C)** HaCaT cells were incubated with either DMSO (−) or increasing concentrations of CBD. Three hours later, cells were collected, nuclear and cytosolic fractions were isolated and the nuclear fraction was analysed for the levels of BACH1 and NRF2. **D)** HaCaT cells were incubated with either DMSO or CBD (10 μM) for different periods of time as indicated. Cells were lysed and protein levels of BACH1 were analysed. **E)** HaCaT cells were incubated with either DMSO (−) or MG132 (10 μM). Two hours later, CBD was added. Three hours later, cells were collected, nuclear and cytosolic fractions were isolated and analysed for the levels of BACH1. Upper panel is a representative western blot; lower panel shows the quantification of BACH1 protein levels in the nuclear and cytosolic fraction normalized against their respective loading controls. Data represent means ± SD (n = 3) and are expressed relative to the DMSO samples. **F)** HaCaT cells were incubated with either DMSO (−) or Leptomycin B (LMB) (5 nM). Two hours later, CBD (10 μM) was added for another 3 h. After that, cells were lysed and protein levels of BACH1 were analysed.

Based on our results, CBD only weakly stabilises NRF2, and its positive effect on NRF2 target genes is restricted to a subset of genes. Previous studies in keratinocytes have shown that while KEAP1 knockdown led to a weak induction of *HMOX1* (2-5-fold induction), BACH1 knockdown strongly induced *HMOX1* (135-fold induction) without affecting other NRF2 target genes (i.e aldo-ketoreductases) [14]. Therefore, BACH1 appeared as a potential molecular target for CBD that could explain the observed selective transcriptional effect. To test this hypothesis, we compared the induction of *HMOX1* in response to CBD in HaCaT cells transfected with either siControl or siBACH1. In agreement with previous reports, BACH1 knockdown by itself strongly induced *HMOX1* levels (mean 51-fold) (Fig. 4A, *upper left panel*). Importantly, CBD induced the expression of *HMOX1* in control cells (mean 58-fold) but its effect was greatly reduced in BACH1 knocked-down cells (mean 10-fold) (Fig. 4A, *upper right panel*), showing the relevance of BACH1 for the effect of CBD on *HMOX1*. Moreover, BACH1 depletion also abolished the induction of *HMOX1* mediated by the BACH1 inhibitor hemin, but not the one mediated by SFN (Fig. S2A), further demonstrating the different mechanism of action used by CDB and hemin in comparison with SFN.

Most described regulatory mechanisms for BACH1 are at post-translational level, affecting either its localisation and/or stability. For instance, the main negative regulator of BACH1, heme, inactivates BACH1 leading to its nuclear exclusion and cytosolic degradation [13,39,40]. To test if CBD had an effect on the levels of BACH1, we exposed HaCaT cells to increasing concentrations of CBD and measured BACH1 protein levels. Our data showed that CBD dramatically reduced both, BACH1 total (Fig. 4B) and BACH1 nuclear levels (Fig. 4C) in a dose dependent manner, and that this effect is obvious as soon as 2 h after CBD treatment (Fig. 4D). Compared with SFN, CBD is a weak inducer of NRF2, but a potent BACH1 inhibitor (Fig. 3E and Fig. 4B). To test whether CBD was inducing BACH1 proteasomal degradation in either the nucleus or the cytoplasm, we pre-treated the cells with or without the proteasome inhibitor MG132 and analysed the effect of CBD on BACH1 nuclear and cytosolic levels (Fig. 4E). Interestingly, CBD treatment led to reduction of nuclear BACH1, which was not recovered by MG132, suggesting that BACH1 is not degraded in the nucleus. On the other hand, CBD treatment in presence of MG132 led to accumulation of cytosolic BACH1. Based on these results we hypothesized that CBD treatment might induce BACH1 nuclear export and its cytosolic degradation. To further test that hypothesis, we treated cells with or without the nuclear export inhibitor leptomycin B in combination with CBD (Fig. 4F). Our results showed that leptomycin B completely abolished the effect of CBD on BACH1 levels. Altogether, our data demonstrate that CBD leads to BACH1 degradation via a mechanism that involves BACH1 nuclear export and cytosolic degradation.

The mechanism by which CBD regulates BACH1 resembles the one shown for the BACH1 inhibitor hemin [13,39,40]. These similarities prompted us to study if CBD could be inhibiting BACH1 by modulating the levels of intracellular heme. Our results showed that CBD did not increased the levels of heme (Fig. S2B), neither was the effect of CBD on either BACH1 protein levels or *HMOX1* mRNA levels impaired by inhibitors of the heme biosynthesis (Figs. S2C and S2D), suggesting that the effect of CBD is independent of heme.

### 3.5 CBD induces *HMOX1* in a NRF2-independent manner

**Fig. 5:**
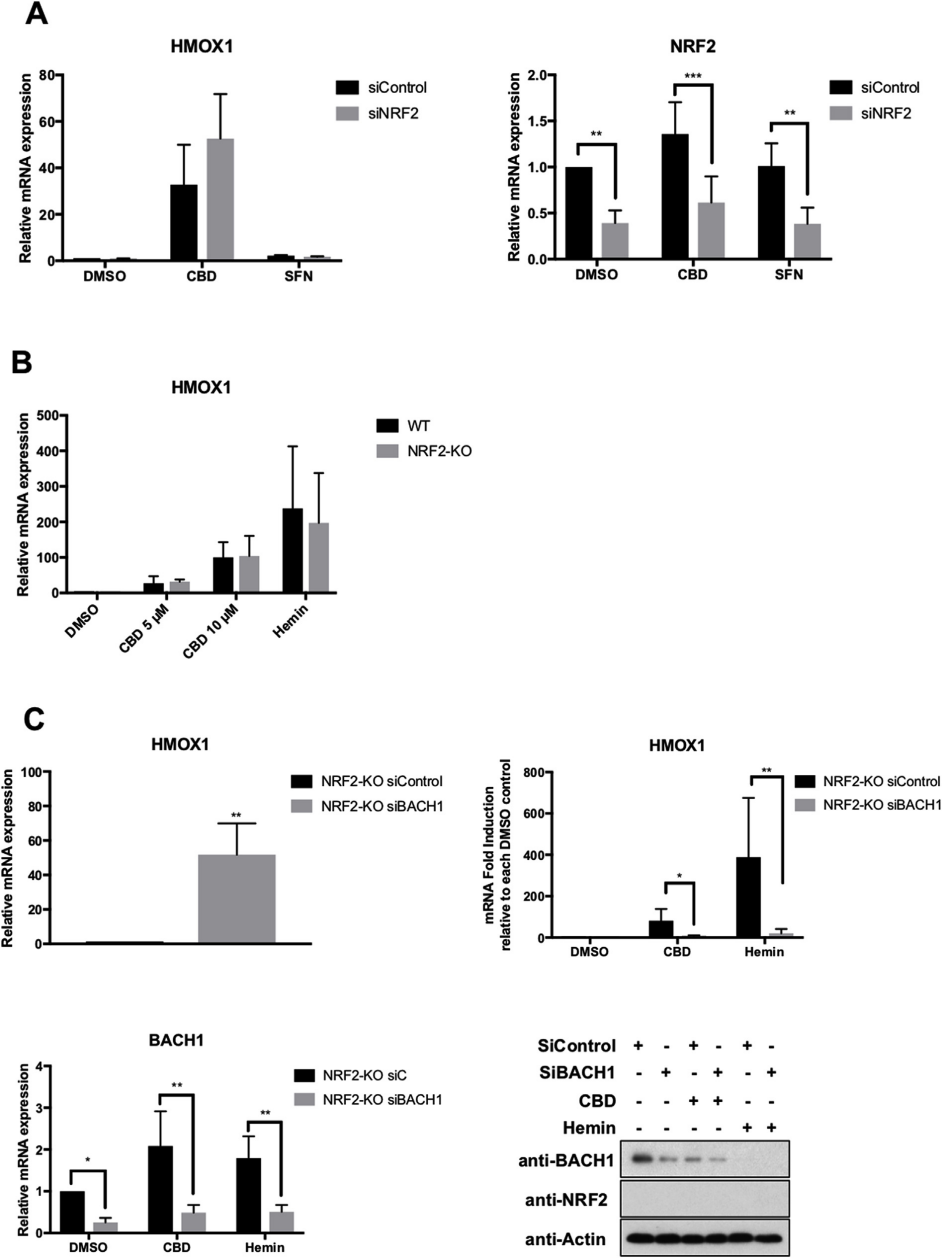
**CBD activates HMOX1 in a NRF2-independent manner. A)** HaCaT cells were transfected with either siControl or siNRF2. 36 h later cells were incubated with either DMSO, CBD (10 μM) or SFN (5 μM) for another 16 h. The mRNA levels for *HMOX1 (left panel)* and *NRF2 (right panel)* were quantified using real-time PCR. The data were normalized using *HPRT1* as an internal control. Data represent means ± SD (n = 3) and are expressed relative to the siControl DMSO sample. **P ≤ 0.01, ***P ≤ 0.001**. B)** Control (WT) and NRF2-KO HaCaT cells were incubated with either DMSO, CBD or Hemin (10 μM). 16 h later, the mRNA levels for *HMOX1* were quantified using real-time PCR as previously described (n = 3). **C)** Control (WT) and NRF2-KO HaCaT cells were transfected with either siControl or siBACH1. 36 h later cells were incubated with DMSO, CBD (10 μM) or hemin (10 μM) for another 16 h. To measure the effect of BACH1 depletion on *HMOX1* expression, the mRNA levels for *HMOX1* in siControl and siBACH1 DMSO-treated cells were quantified using real-time PCR as previously described *(upper left panel)*. To compare the *HMOX1* induction upon treatment with CBD or hemin in each cell line, the levels of *HMOX1* in either siControl CBD/hemin or siBACH1 CBD/hemin were compared against the levels of *HMOX1* in either siControl DMSO or siBACH1 DMSO respectively (*HMOX1* levels in DMSO samples were set in both cases as 1) *(upper right panel*). The *HMOX1* induction in response to either CBD or hemin was quantified by real-time PCR using *HPRT1* as an internal control. Data represent means ± SD (n = 3). To control for the efficiency of the knockdown, the mRNA levels *(lower left panel)* and protein levels *(lower right panel)* of BACH1 were analysed by real-time PCR and western blot respectively. *P ≤ 0.05, **P ≤ 0.01.

In the current model, BACH1 depletion is the first step before NRF2 could induce *HMOX1*. Therefore, we hypothesized that as the effect of CBD on *HMOX1* involved BACH1 inhibition, it would be impaired by NRF2 depletion. Surprisingly, NRF2 knockdown did not affect CBD-mediated *HMOX1* induction (Fig. 5A) although, it impaired the SFN-mediated *AKR1C1* induction (Fig. S3A). To confirm our results using a complementary approach, we produced CRISPR-mediated NRF2-KO HaCaT cells. We verified that these cells had no detectable NRF2 protein levels (Fig. S3B), and significantly lower mRNA levels of *NQO1* and *AKR1B10*, two of the best markers for NRF2 activity (Fig. S3C). Using these cells, we found that in agreement with our siRNA approach, both CBD and hemin were still able to strongly induce *HMOX1* in the absence of NRF2 (Fig. 5B), while the effect of SFN was completely abolished (Fig. S3D), suggesting that NRF2 is not necessary for *HMOX1* induction mediated by BACH1. However, as both compounds, CBD and hemin, are likely to target other proteins in addition to BACH1, their effect on *HMOX1* might not be solely dependent on BACH1. To test this possibility, we directly assessed the effect of knocking down BACH1 on *HMOX1* levels in NRF2-KO cells, with or without CBD or hemin treatment. BACH1 knockdown by itself led to a strong induction of *HMOX1* in NRF2-KO HaCaT cells (mean 50-fold) (Fig. 5C, *upper left panel*), similar to the induction observed in WT HaCaT cells (Fig. 4A), showing that NRF2 is not necessary for the *HMOX1* induction upon BACH1 depletion. Additionally, both CBD and hemin strongly induced *HMOX1* in NRF2-KO HaCaT cells, and BACH1 depletion almost completely impaired the induction of *HMOX1* mediated by both compounds (Fig. 5C, *upper right panel*). These results demonstrate that: a) the effect of CBD and hemin on *HMOX1* expression is BACH1-dependent and NRF2-independent; and b) in HaCaT cells, NRF2 is not necessary for BACH1-mediated *HMOX1* induction.

### 3.6 Effect of topical CBD in skin *in vivo*

**Fig. 6:**
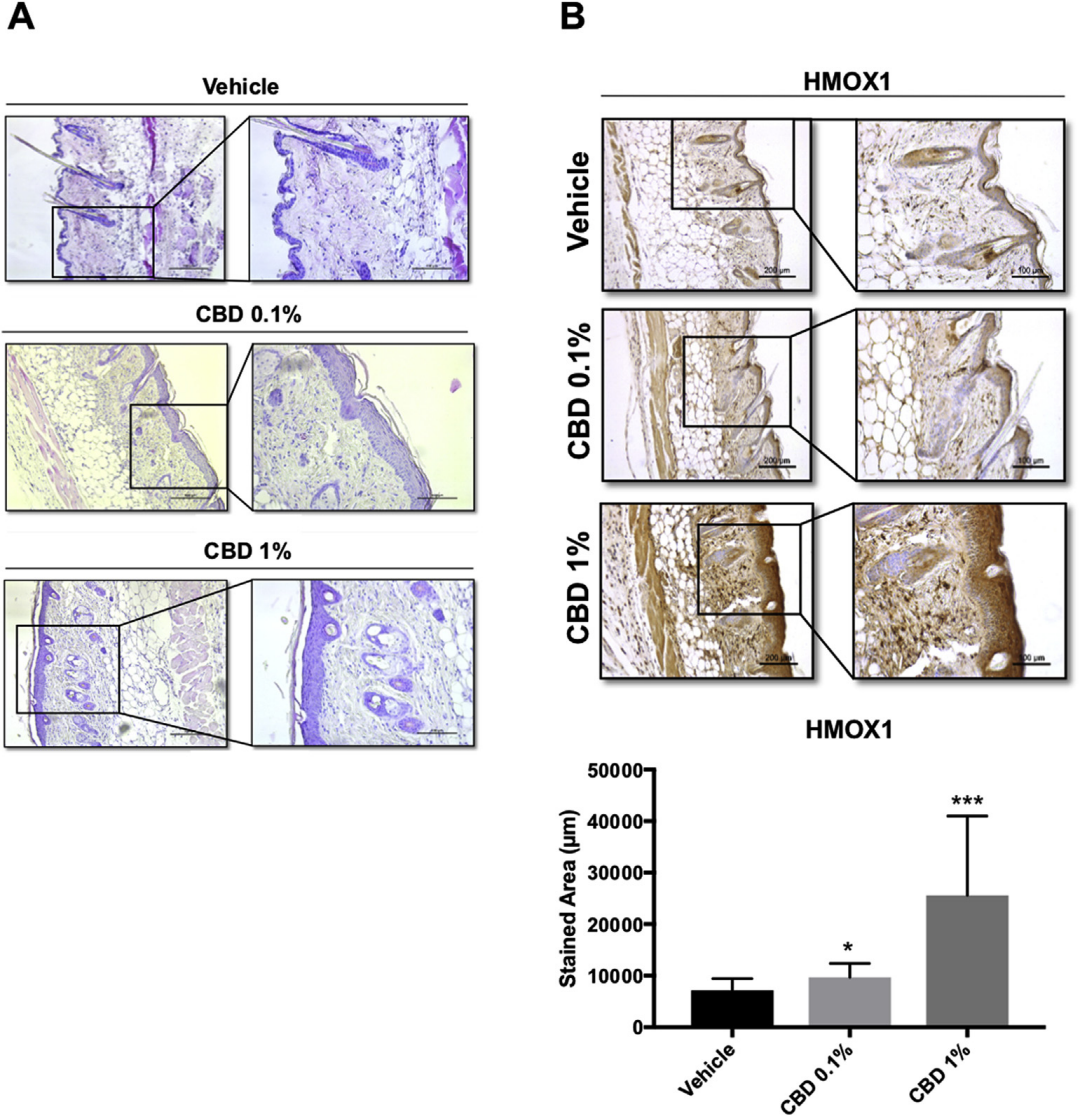
**CBD treatment increases the keratinocyte layer in the epidermis and the expression of HMOX1. (A)** Haematoxylin-eosin staining of 5 μm paraffin-embedded sections were analysed by bright field microscopy. **(B)** Representative images of HMOX1 immunohistochemistry from mouse skin after 5 days of treatment with vehicle, CBD 0.1% or 1% **(C)** Quantification of HO-1 stained area in mouse epidermis. *P < 0.05, ***P < 0.001 compared with control. Scale bars: 200 (left) and 100 μm (right). n = 15 for vehicle treated samples and n = 12 for CBD treated samples.

**Fig. 7:**
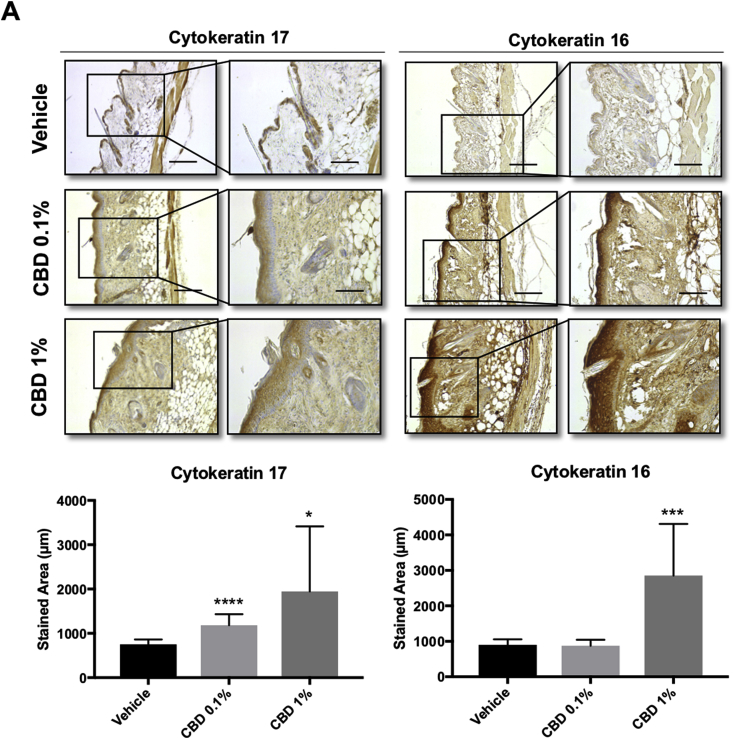
**CBD treatment significantly increases the expression of cytokeratin 17 and 16 in mouse epidermis. (A)** Representative images of cytokeratin 17 and 16 immunohistochemistry from mouse skin after 5 days of treatment with vehicle, CBD 0.1% or 1% **(B)** Quantification of cytokeratin 17 and 16 stained areas in mouse epidermis. *P < 0.05, ***<0.001 compared with control. Scale: 200 (left) and 100 μm (right). n = 14 for vehicle treated samples and n = 10 for CBD treated samples.

Next, we treated mice with concentrations of topically-applied CBD that mimic the range of concentrations found in commercially available CBD-based products (0.1–1%) and studied the effect of CBD on epidermal morphology. As we are interested in the effect of CBD on the skin of adults (which is the age group for which CBD-based creams is marketed), we used 6 months old mice, a model that resembles adult skin. We observed that treatment with CBD induced keratinocyte proliferation (measured by epidermal thickness) (Fig. 6A) and increased the levels of HMOX1 (Fig. 6B). Moreover, CBD also increased the levels of cytokeratins 16 and 17 (Fig. 7A), which are associated with keratinocyte hyperproliferation [41] and wound repair [42]. Although both HMOX1 and KRT16 and KRT17 are also considered markers of stress and inflammation, CBD did not induce the expression of typical pro-inflammatory cytokines (i.e IL1β, IL6 and TNFα) (Fig. S4) suggesting that the effect of CBD on *HMOX1* is not due to an inflammatory response.

## 4 Discussion

The potential use of CBD for treatment of skin disorders and cosmetic indications is gaining momentum, although the mechanism of action of CBD on different skin cell types is not understood [43,44]. Using a system biology approach combining transcriptomic and proteomic data we have identified for the first time the major pathways that are regulated by CBD on human primary keratinocytes cultivated under non-differentiating conditions, which could explain some of the potential beneficial effects of CBD in the skin. Importantly, although a potential link between CBD and the NRF2 pathway has been suggested in other cells types, no molecular mechanism has been identified so far. In that sense, our work reveals for the first time the link connecting CBD with the NRF2 pathway. However, although our study does not identify the specific molecular mechanism by which CBD induces BACH1 cytosolic degradation, our results suggest that it is independent on the levels of heme, and thus a different mechanism than the one used by the BACH1 inhibitor Hemin. A recent report showed that Fbxo22 is the E3 ligase responsible for the degradation of BACH1 [45]. Due to the rapid effect of CBD on BACH1 levels, we hypothesise that CBD might be directly affecting the same or a different E3 ligase controlling BACH1 turnover.

It is interesting that although one of the signatures identified in our system biology analysis was the NRF2 pathway, which suggested that CBD could be an NRF2 activator, our biochemical analysis demonstrated that CBD is a weak NRF2 activator but a potent BACH1 inhibitor, and thus BACH1 is the main target of CBD in keratinocytes. As the transcriptional signature of BACH1 overlaps with that of NRF2, it is not unexpected that a BACH1 inhibitor presents a signature partially similar to an NRF2 activator. Nevertheless, our analysis shows that CBD is also a weak inducer of NRF2, which could be an indirect effect of its negative effect on BACH1. A potential explanation could be that as BACH1 repress p62 expression, and CBD inhibits BACH1 increasing p62 levels, CBD might stabilise NRF2 indirectly by inducing the expression of the KEAP1 competitor p62 [46].

The best characterised BACH1 target is *HMOX1*, which is the mRNA and the protein that was most regulated by CBD in our analyses. Unexpectedly, we identified that the *HMOX1* induction mediated by CBD was NRF2 independent. In the current general model of *HMOX1* activation, BACH1 and NRF2 work together controlling its expression. However, pioneer work in mice showed that although that is true for some tissues (i.e. lung, heart and liver), in thymus the induction of HMOX1 in response to BACH1 depletion was independent of NRF2 [47]. Our results demonstrate that in HaCaT cells, NRF2 is not necessary for the induction of *HMOX1* upon BACH1 depletion/inhibition, supporting a novel regulatory mechanism involving BACH1 and an unidentified positive regulator. This agrees with the original publication, showing that the role of NRF2 controlling HMOX1 expression might be redundant with other activators in a tissue/cell line specific manner. Although NRF2 is the main positive regulator, *HMOX1* expression can also be regulated by other members of the CNC (NRF1 and NRF3) and MAF family (large and small MAFs), HSF1, c-jun, AP1 and NF-κB [48], and thus either of these factors could be responsible for the induction of *HMOX1* in response to BACH1 depletion/inhibition. As CBD induces the expression of a MAFB expression signature (Fig. 1D) and also of small *MAFF* and *MAFG* (Fig. 2B), these members of the MAF family could potentially be involved in the positive regulation of *HMOX1* in the absence of BACH1. Further work is necessary to address which factors can compensate for the lack of NRF2 in the different tissues, and in HaCaT and primary keratinocytes in particular.

In skin, HMOX1 is an important cytoprotective enzyme with antioxidant, anti-inflammatory and anti-apoptotic properties. Due to such protective roles, treatments that regulate *HMOX1* expression would be useful for the treatment of inflammatory- or oxidative stress-associated skin conditions. In these scenarios, BACH1 inhibitors might be very useful, due to their potent activity as *HMOX1* inducers. Our validation of CBD as an BACH1 inhibitor suggests that CBD treatment would a) protect the skin against external insults: e.g. against UVA-irradiation-induced damage; and b) be greatly beneficial in a variety of skin conditions, e.g. eczema or atopic dermatitis.

Previous studies have shown that CBD inhibited differentiation in HaCaT cells [49], and exerted anti-proliferative actions on transformed human keratinocytes [50]. Our system analysis *in vitro* showed an anti-proliferative and pro-differentiation profile for CBD, however, our *in vivo* data showed that CBD induced keratinocyte proliferation measured by an increment in both skin thickness and in the levels of the proliferative keratins K16 and K17. HMOX1 has been suggested to induce keratinocyte proliferation [51], which could explain our results *in vivo*, although we cannot rule out the participation of additional proliferative factors regulated by CBD.

Anectodical evidence suggested that CBD could be indicated for the treatment of psoriatic plaques. However, psoriasis is characterized by chronic inflammation and keratinocyte hyperproliferation (i.e with high levels of keratin 16 and 17); therefore, and although CBD has anti-inflammatory effects, we consider that the use of CBD in psoriasis should be taken with caution due to its pro-proliferative effects *in vivo*. Further experiments are required to determine the effect of CBD treatment in psoriatic lesions *in vivo.*

The NRF2 activator SFN has been shown to be beneficial for keratin disorders such as Epidermolysis Bullosa Simplex (EBS). In this context, topical treatment with SFN rescued skin blistering in an EBS mouse model, correlating with the reprogramming of keratin synthesis in epidermis and induction of keratin 16 and 17 [52]. Curiously however, whereas the SFN-mediated induction of keratin 16 partly depends on NRF2, the induction of keratin 17 is NRF2-independent [53]. Since topical and oral CBD have been used by EBS patients with promising results [22,54] our results provide a mechanistic explanation and support CBD as a promising option for the prevention of the pathological skin fragility occurring in EBS and for improving wound closure.

Regarding the safety used of BACH1 inhibitors, long-term BACH1 deficiency and associated sustained *HMOX1* upregulation in *Bach1*^−/−^ mice did not show any detrimental effect under normal conditions [55], which suggest that acute BACH1 inhibition would not have any systemic deteriorative effect under such conditions.

In summary our study demonstrates for the first time a biochemical target for CBD and provides a scientific rationale for its use as a treatment for some skin conditions. Furthermore, recent reports have highlighted the potential value of BACH1 as a therapeutic target in cancer [45,56,57], and thus our study also opens the door for new lines of investigation focused on the potential value of CBD as a cancer therapeutic drug.

## Funding

This work was supported by the Medical Research Institute of the University of Dundee, Cancer Research UK (C52419/A22869) (LV) and Tenovus Scotland (T18/07) (LC) and by grant SAF2017-87701-R (EM) from the Ministry of the Economy and Competition (MINECO) co-financed with the European Union FEDER funds. InnoHealth Group and Emerald Health Biotechnology also supported this work and had no further role in study design, the collection, analysis and interpretation of data, in the writing of the report, or in the decision to submit the paper for publication.

## Authors contribution

LC, VG, EM and JAC conducted the *in vitro* experiments, the histopathology and the immuno-histochemistry studies. VG coordinated and conducted, together with AGM and JP, the whole set of *in vivo* experiments, as well as different of the analytical procedures related with those. MGR and MAC conducted the bioinformatic analysis. LC, VG, MGR, EM and JP were responsible for initial data analysis, figure preparation and statistical analysis. LV and EM had a leading contribution in the design of *in vivo* studies, and an active role in the discussion and interpretation of the whole dataset. LV and EM jointly wrote the manuscript as well as by the rest of the authors. All the authors take full responsibility for the work.

## Declaration of interests

Eduardo Muñoz is a member of the Scientific Advisory Board of InnoHealth Group and Emerald Health Biotechnology.
