# Supplementary material for: Cannabidiol induces antioxidant pathways in keratinocytes by targeting BACH1
Source: Redox Biol. 2019 Sep 5;28:101321. doi: 10.1016/j.redox.2019.101321 (PMC6742916; doi:10.1016/j.redox.2019.101321)
Supplement: Multimedia component 1 [file mmc1.pdf]

SUPPLEMENTARY MATERIAL

Supplementary Table 1: Primers used in this study:

**H-HMOX1 f**	5′-CTCAAACCTCCAAAAGCC-3′
**H-HMOX1 r**	5′-TCAAAAACCACCCCAACCC-3′
**H-SQSTM1 f**	5′- CTGGGACTGAGAAGGCTCAC-3′
**H-SQSTM1 r**	5′-GCAGCTGATGGTTTGGAAAT-3′
**HPRT1**	Hs02800695_m1 HPRT1 TaqMan (s)
**HMOX1**	Hs01110250_m1 HMOX1 TaqMan (s)
**NQO1**	Hs01045993_g1 NQO1 TaqMan (m)
**AKR1C1**	Hs01061917_g1 SQSTM1 TaqMan (s)
**AKR1B10**	Hs00252524_m1 AKR1B10 TaqMan (s)
**SQSTM1**	Hs01061917_g1 SQSTM1 TaqMan (s)
**IL-1β f**	5´-CTCCACCTCAATGGACAGAA-3´
**IL-1β r**	5´-GCCGTC TTTCATTACACAGG-3´
**TNFα f**	5´-CTACTCCCAGGTTCTCTTCAA-3´
**TNFα r**	5´-GCAGAGAGGAGGTTGACTTTC-3´
**IL-6 f**	5´-GTATGAACAACGATGATGCACTTG-3´
**IL-6 r**	5´-ATGGTACTCCAGAAGACCAGAGGA-3´
**KRT17 f**	5´-GATGGAGCAGAACCAGGAGTAC-3´
**KRT 17 r**	5´-GGTCTCAAGCATAGGAATGCTGGGG-3´

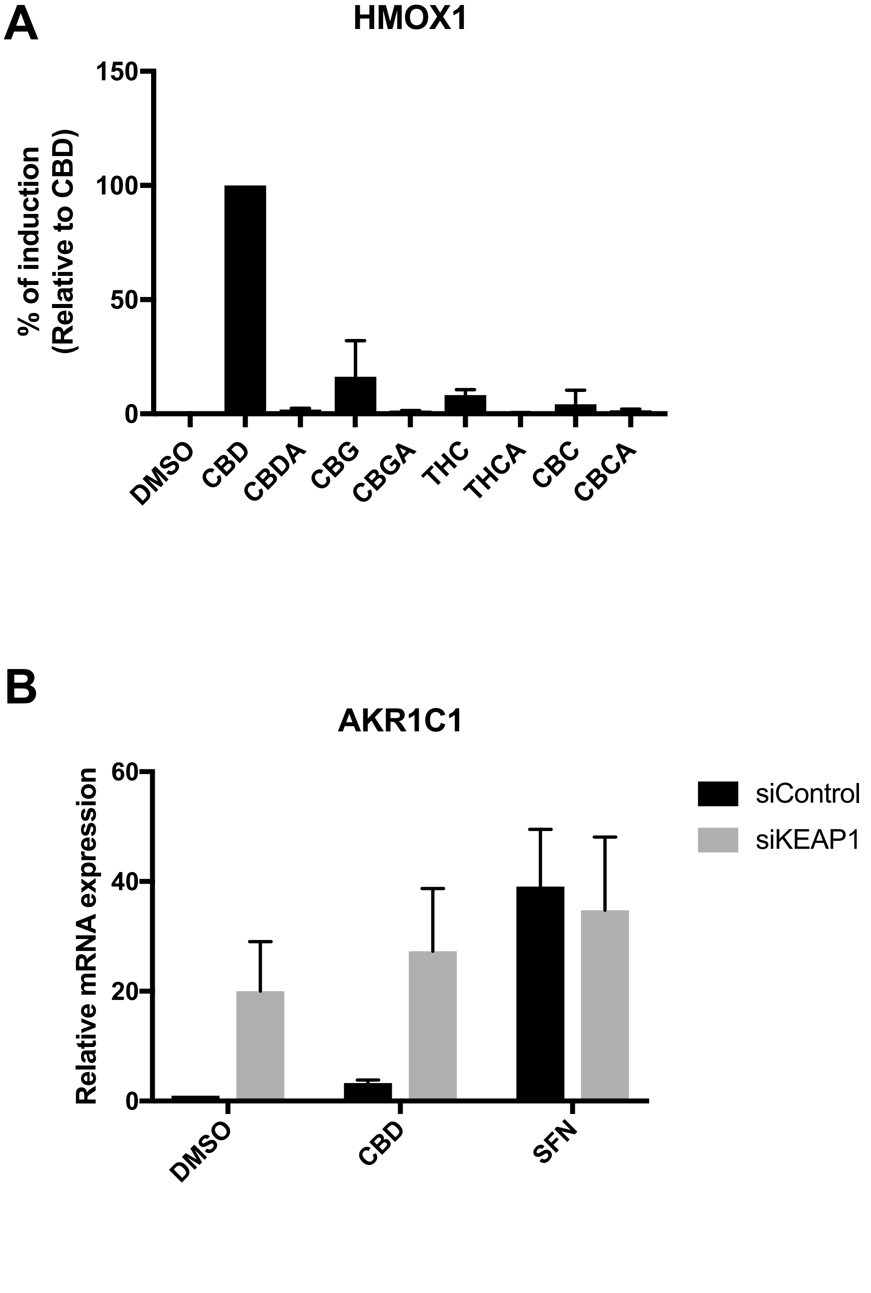

Figure S1. A) HaCaT cells were incubated with either DMSO, CBD (10 µM) or the indicated cannabinoids (10 µM). 16 hours later the mRNA levels for *HMOX1* were quantified as previously described (n=3). **B)** HaCaT cells were transfected with either siControl or siKEAP1. 36 hours later cells were incubated with either DMSO, SFN (5 µM), or CBD (10 µM) for another 16 hours. The mRNA levels for *AKR1C1* were quantified as previously described (n=3).

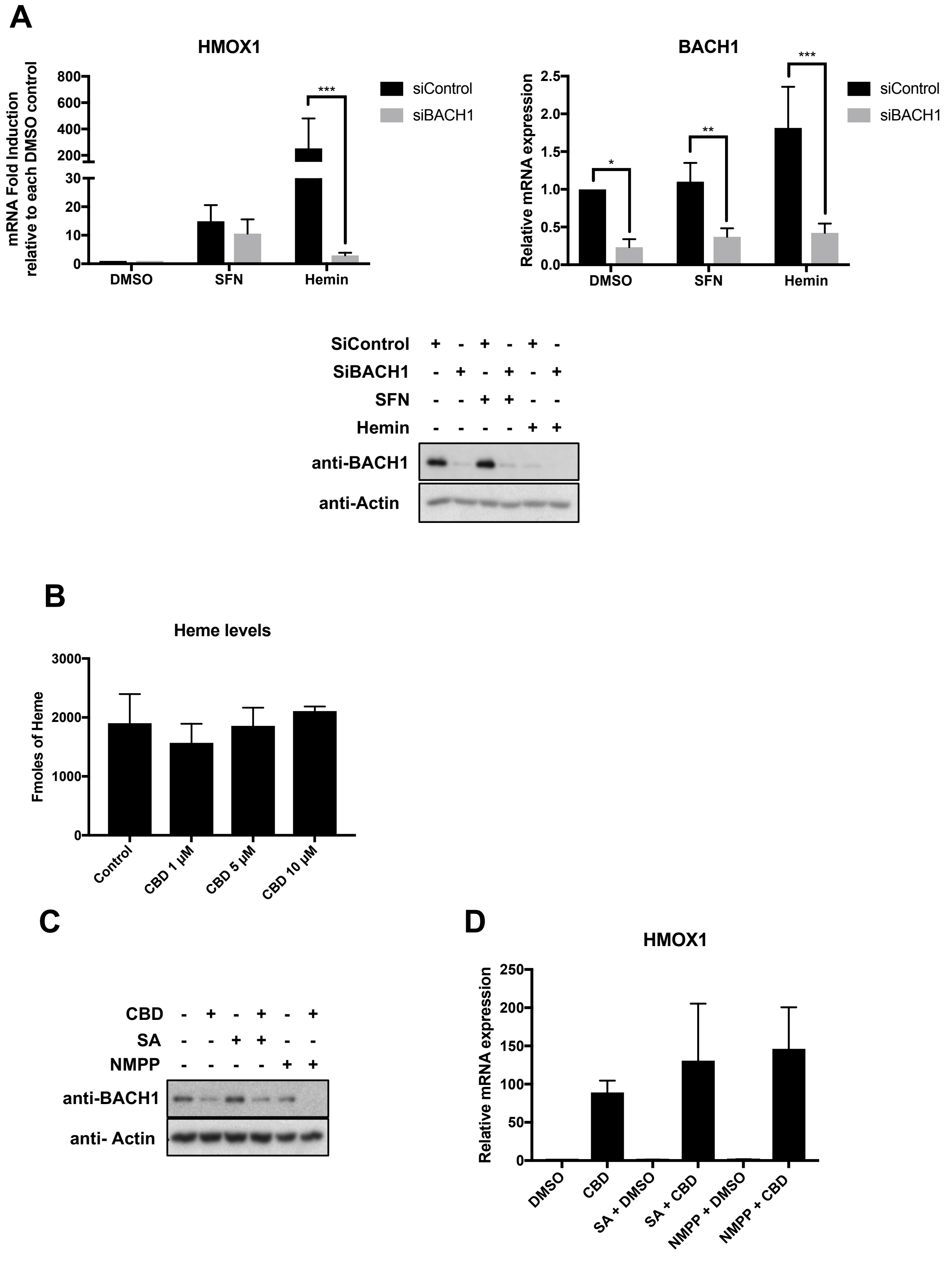

Figure S2. A) HaCaT cells were transfected with either siControl or siBACH1. 36 hours later cells were incubated with either DMSO, SFN (5 µM) or Hemin (10 µM) for another 8 hours. The mRNA levels for *HMOX1* were quantified by real-time PCR using *HPRT1* as an internal control. Data represent means ± SD (n=3). To compare the *HMOX1* induction upon treatment in each cell line, the levels of *HMOX1* in each siRNA treated cell line (either siControl SFN/Hemin or siBACH1 SFN/Hemin) was compared against the levels of *HMOX1* in their

own DMSO control sample (set in both cases as 1) (i.e. either siControl DMSO or siBACH1 DMSO respectively) *(upper leftt panel*). To control for the efficiency of the knockdown, the mRNA levels *(upper right panel)* and protein levels *(lower panel)* of BACH1 were analyzed by real-time PCR and western blot respectively**.** *P ≤ 0.05, **P ≤ 0.01, ***P ≤ 0.001, ****P≤ 0.0001. **B)** HaCaT cells were treated with increasing concentrations of CBD for 3 h and the levels of Heme detected in the supernatants. **C)** HaCaT cells were incubated with either DMSO, Succinylacetone (SA) (1 mM) or N-methyl Protoporphyrin IX (NMPP) (20 μM). 36 hours later CBD (10 μM) was added. Three hours later cells were lysed and levels of BACH1 were analysed by western blot. **D)** HaCaT cells were incubated with either DMSO, Succinylacetone (SA) (1 mM) or N-methyl Protoporphyrin IX (NMPP) (20 μM). 36 hours later CBD (10 μM) was added. 16 hours later the mRNA levels for *HMOX1* were quantified using real-time PCR as previously described (n=3).

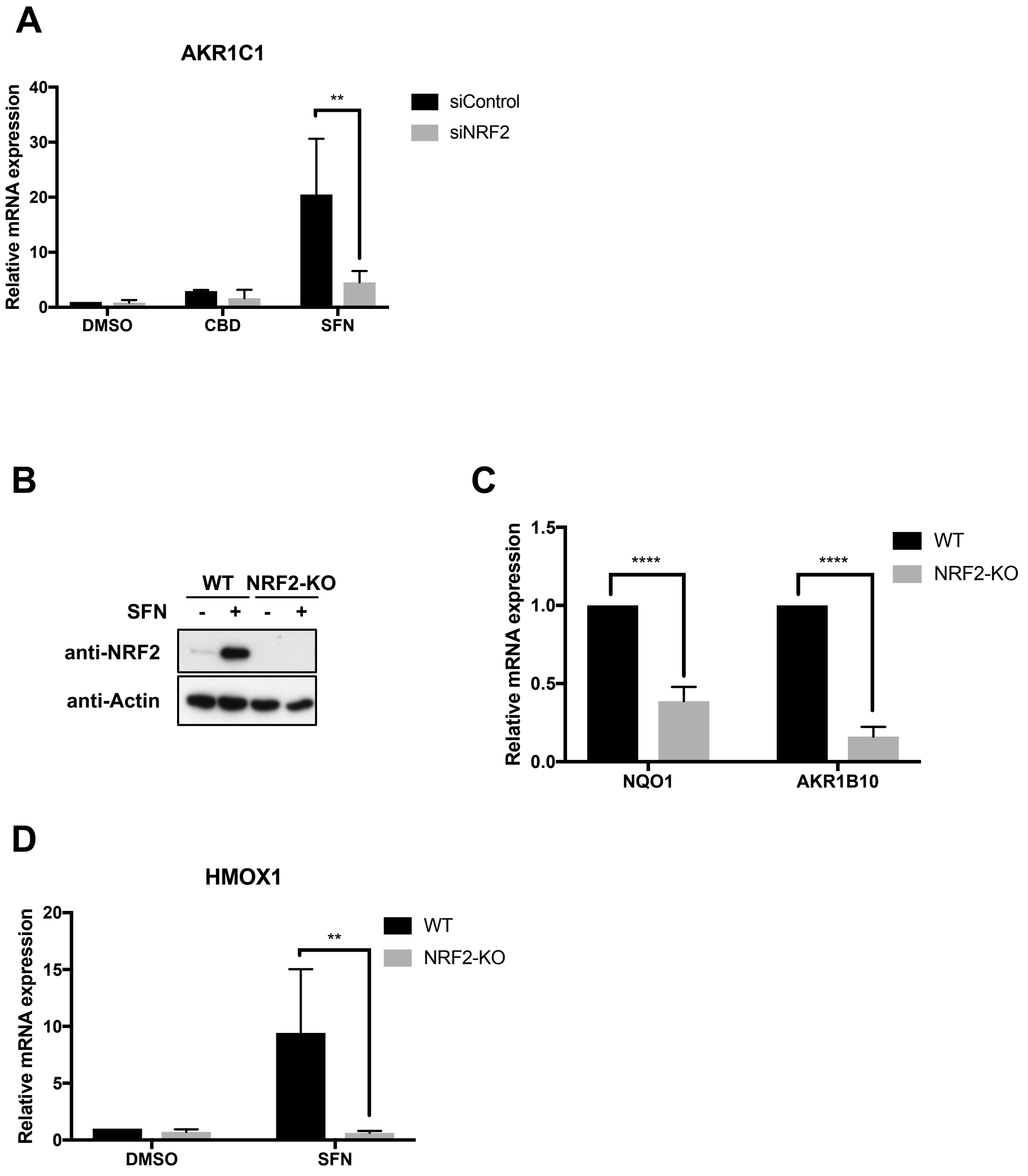

Figure S3. **A)** HaCaT cells were transfected with either siControl or siNRF2. 36 hours later cells were incubated with either DMSO, SFN (5 µM) or CBD (10 µM) for another 16 hours. The mRNA levels for *AKR1C1* were quantified using real-time PCR. The data were normalized using HPRT1 as an internal control. Data represent means ± SD (n=3) and are expressed relative to the siControl DMSO sample. **P ≤ 0.01. **B)** Control (WT) and NRF2-KO HaCaT cells were incubated with either DMSO (-) or SFN (5 µM). Three hours later, cells were lysed and the levels of NRF2 were analyzed by western blot. **C)** Control (WT) and NRF2-KO HaCaT cells were were collected and the mRNA levels for *NQO1* and *AKR1B10* were quantified using real-time PCR as previously described (n=3). **D)** Control (WT) and NRF2-KO HaCaT cells were incubated with either DMSO (-) or SFN (5 µM). Eight hours later, the mRNA levels for *HMOX1* were quantified using real-time PCR as previously indicated (n=3)

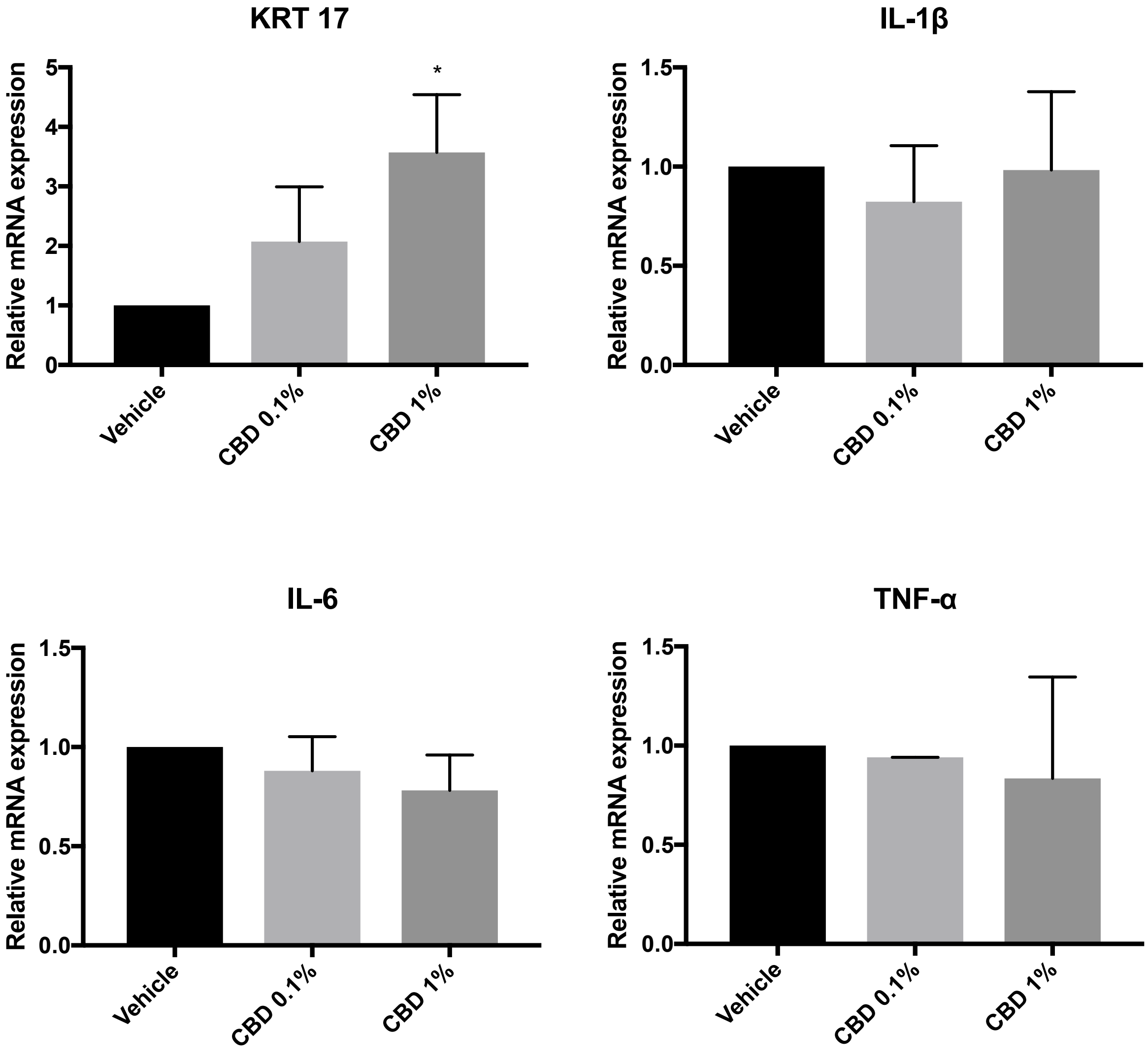

Figure S4. The mRNA levels for Keratin 17 (*KRT17), Interleukin 1 beta (IL1β), Interleukin 16 (IL-6)* and *Tumour Necrosis Factor alpha (TNFα)* in mouse skin after 5 days of treatment with vehicle, CBD 0.1% or CBD 1% were quantified using real-time PCR. The data were normalized using HPRT1 as an internal control. Data represent means ± SD (n=3) and are expressed relative to treatment with vehicle. *P ≤ 0.05.
